# Developing a New qFIBS Model Assessing Histological Features in Pediatric Patients With Non-alcoholic Steatohepatitis

**DOI:** 10.3389/fmed.2022.925357

**Published:** 2022-06-27

**Authors:** Feng Liu, Lai Wei, Wei Qiang Leow, Shu-Hong Liu, Ya-Yun Ren, Xiao-Xiao Wang, Xiao-He Li, Hui-Ying Rao, Rui Huang, Nan Wu, Aileen Wee, Jing-Min Zhao

**Affiliations:** ^1^Peking University People's Hospital, Peking University Hepatology Institute, Beijing Key Laboratory of Hepatitis C and Immunotherapy for Liver Diseases, Beijing International Cooperation Base for Science and Technology on NAFLD Diagnosis, Beijing, China; ^2^Hepatopancreatobiliary Center, Beijing Tsinghua Changgung Hospital, Tsinghua University, Beijing, China; ^3^Department of Anatomical Pathology, Singapore General Hospital, Singapore, Singapore; ^4^Duke-NUS Medical School, Singapore, Singapore; ^5^Department of Pathology and Hepatology, The Fifth Medical Center of PLA General Hospital, Beijing, China; ^6^HistoIndex Pte Ltd., Singapore, Singapore; ^7^Department of Pathology, Yong Loo Lin School of Medicine, National University of Singapore, National University Hospital, Singapore, Singapore

**Keywords:** automated quantitative evaluation, fibrosis, inflammation, ballooning, steatosis, liver, pediatric NASH

## Abstract

**Background:**

The evolution of pediatric non-alcoholic fatty liver disease (NAFLD) to non-alcoholic steatohepatitis (NASH) is associated with unique histological features. Pathological evaluation of liver specimen is often hindered by observer variability and diagnostic consensus is not always attainable. We investigated whether the qFIBS technique derived from adult NASH could be applied to pediatric NASH.

**Materials and Methods:**

102 pediatric patients (<18 years old) with liver biopsy-proven NASH were included. The liver biopsies were serially sectioned for hematoxylin-eosin and Masson trichrome staining for histological scoring, and for second harmonic generation (SHG) imaging. qFIBS-automated measure of fibrosis, inflammation, hepatocyte ballooning, and steatosis was estabilshed by using the NASH CRN scoring system as the reference standard.

**Results:**

qFIBS showed the best correlation with steatosis (*r* = 0.84, *P* < 0.001); with ability to distinguish different grades of steatosis (AUROCs 0.90 and 0.98, sensitivity 0.71 and 0.93, and specificity 0.90 and 0.90). qFIBS correlation with fibrosis (*r* = 0.72, *P* < 0.001) was good with high AUROC values [qFibrosis (AUC) > 0.85 (0.85–0.95)] and ability to distinguish different stages of fibrosis. qFIBS showed weak correlation with ballooning (*r* = 0.38, *P* = 0.028) and inflammation (*r* = 0.46, *P* = 0.005); however, it could distinguish different grades of ballooning (AUROCs 0.73, sensitivity 0.36, and specificity 0.92) and inflammation (AUROCs 0.77, sensitivity 0.83, and specificity 0.53).

**Conclusion:**

It was demonstrated that when qFIBS derived from adult NASH was performed on pediatric NASH, it could best distinguish the various histological grades of steatosis and fibrosis.

## Introduction

Non-alcoholic fatty liver disease (NAFLD) is now the most common chronic liver disease worldwide (1), with potentially serious health effects not only for adults but also for children (2–5). There are very few epidemiological data on pediatric NAFLD—the overall prevalence of the disease is estimated to be 7.6% in the general pediatric population and 34.2% in the obese population (3). In China, (5) it is estimated that 45% of adolescents with obesity have fatty liver disease. The increasing prevalence of pediatric NAFLD is closely associated with the potential for progression to advanced liver disease/cirrhosis and cardiometabolic syndrome in adulthood, resulting in a significant economic burden to society. However, to date, no treatment has been registered for NAFLD for either adults (6) or children (7), and as of now, the treatment of pediatric NAFLD represents a major challenge.

The limitations of clinical trials performed for pediatric NAFLD include the lack of standardization in the diagnostic criteria used and the inconsistently defined outcomes. Various non-invasive biomarkers have been used to identify NAFLD, including traditional imaging techniques such as ultrasound, ultrasound-based elastography, computed tomography (CT), magnetic resonance spectroscopy (MRS), or magnetic resonance elastography (MRE) (8, 9), as well as blood biomarkers (10) such as alanine or aspartate aminotransferase levels (ALT or AST) or diagnostic models (11). However, none have been reliable or qualified as clinical trial endpoints, and none can accurately evaluate grades of steatosis, hepatocyte ballooning, inflammation or fibrosis. Liver biopsy is considered a good choice for accurate diagnosis of NAFLD and monitoring of disease progression during treatment; it also has the additional value of excluding other liver diseases mimicking NAFLD.

To indicate the severity and progression of NAFLD, numerical scores can be assigned to the grades of activity and stages of fibrosis to convey a quantitative assessment of the histological features. Pathologically, the NAFLD activity score (NAS) Clinical Research Network (CRN) scoring system (12), widely used for scoring in clinical trials, can score the features of NAFLD, namely, steatosis (0–3), lobular inflammation (0–3), hepatocyte ballooning (0–2), and fibrosis (0–4). But there are still concerns about potential interobserver variability and the lack of clear diagnostic consensus (13–16). In addition, the current scoring system can only carry out non-linear semiquantitative or classified evaluation of diseases. This may limit the accuracy and granularity of the data, especially in the case of minor changes in treatment. Therefore, standardized and continuous quantitative scales are needed to perform better intuitively, especially when measuring interval changes and treatment responses.

Recently, a number of digital imaging analysis techniques demonstrated the feasibility of assessing liver histological features in NAFLD patients, and may provide automated and quantitative results based on a continuous arithmetic scale (17–21). Taylor-Weiner et al. (19) investigated the application of artificial intelligence tools in quantifying treatment response of patients with NAFLD—their model could characterize disease severity and sensitively quantify treatment response in NASH. In contrast to multiple studies in the adult population, there are no data currently available on the usefulness of digital imaging analysis for the evaluation of pediatric NASH pathology or monitoring of the response to drugs used to treat NAFLD or NASH in children. Our previous research (18) first established a novel second harmonic generation (SHG)/two-photon excitation fluorescence (TPEF)-based automated quantitative evaluation tool, termed “qFIBS,” for the assessment of the four main histological features of NASH in adult patients. However, the histological patterns in adult and pediatric NAFLD usually differ; (22–24) with a pediatric propensity for zone 1 steatosis, inflammation, and fibrosis. Therefore, we aim to further explore the utility and potential of qFIBS in pediatric NASH, with the goal of increasing the diagnostic precision to complement the role of liver pathologists.

## Materials and Methods

### Patients

A total of 102 biopsies from pediatric patients younger than 18 years with biopsy-proven NASH were collected from two centers (Peking University People's Hospital and the Fifth Medical Center of PLA General Hospital, China). Other causes of chronic liver disease, including alcoholic or drug-induced liver disease, autoimmune liver disease, viral hepatitis, and cholestatic or genetic liver diseases, were excluded. This study was approved by the Ethics Committees of both hospitals (No. 2017PHB133-01). Written consent for all participants was obtained from a parent or legal guardian, and assent from all children 5 years and older was obtained before participation.

### Tissue Preparation

Liver biopsies, which were routinely ≥ 10 mm in length, were serially sectioned at 4 μm thickness for SHG imaging and hematoxylin-eosin and Masson trichrome staining for histological scoring. Liver histology from all cases was evaluated by three expert liver pathologists (AW, WQL, and JMZ) based on the NASH CRN scoring system (steatosis: 0–3, ballooning: 0–2, and lobular inflammation: 0–3) (12). NASH was defined by the presence of at least 5% hepatic steatosis with lobular inflammation and definite ballooning, with or without fibrosis. Fibrosis was staged from 0 to 4. Clinical characteristics were obtained from the patients' medical records.

### Image Acquisition

All sample imaging and processing by the imaging system were performed as previously described (18). Briefly, SHG, and TPEF microscopy were utilized to visualize collagen and the histological structures (inflammation, steatosis, and hepatocyte ballooning), respectively. The samples were laser-excited at 780 nm. SHG and TPEF signals were recorded at 390 nm and 550 nm, respectively. The SHG/TPF images for the whole tissue samples were acquired by stitching the adjacent image tiles. The resolution for each image tile is 512 x 512 pixels with dimensions of 200 x 200 μm^2^.

### Training and Validation of the qFIBS Models

The parameters for liver fibrosis, inflammation, hepatocyte ballooning and steatosis were defined and measured as stated in the Methods section of our previous publication for the adult cohort (18). A total of 128 fibrosis parameters, 63 inflammation parameters, 39 ballooning parameters, and 45 steatosis parameters were measured for each sample.

**Figure 1:**
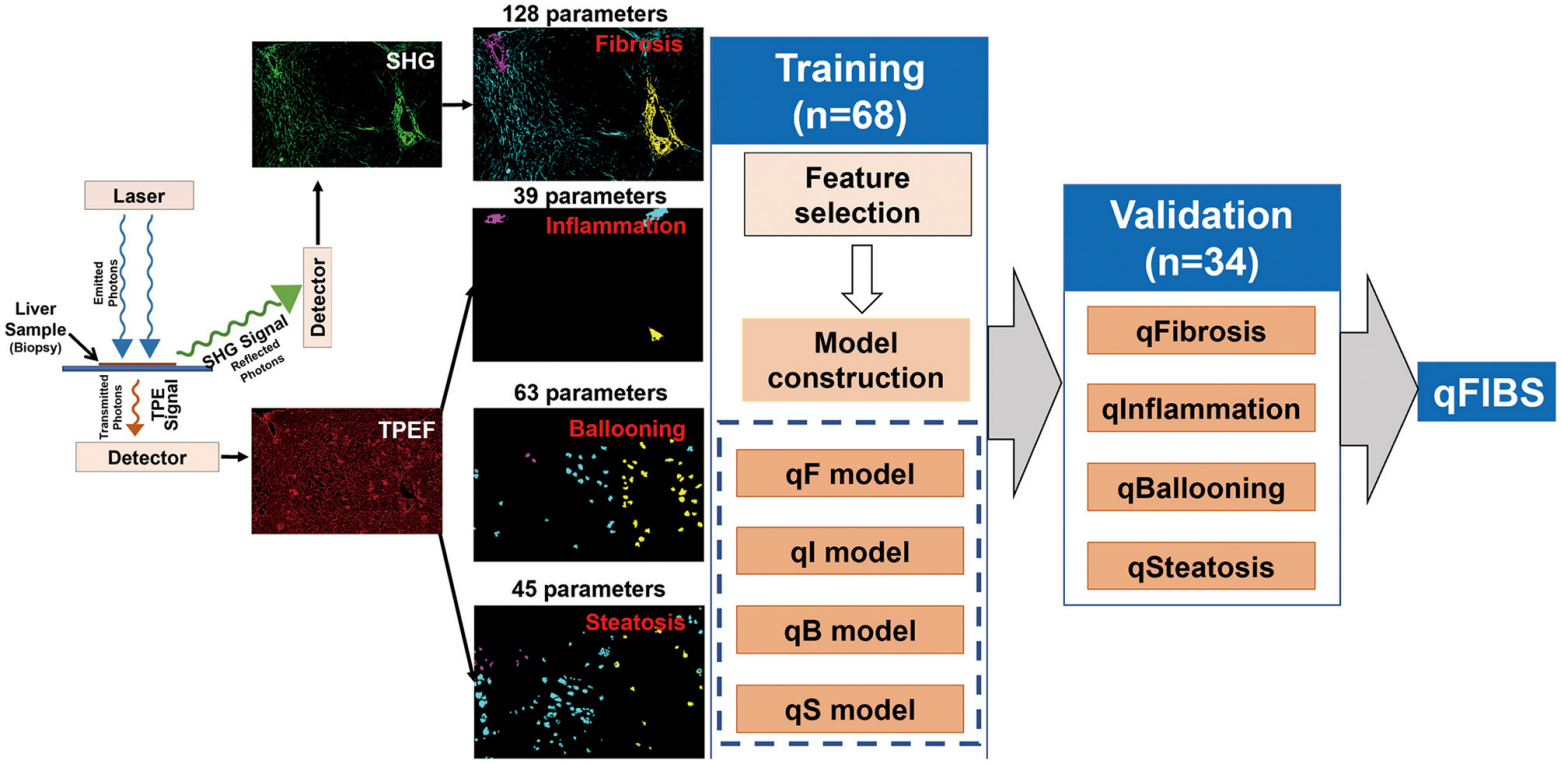
Flowchart shows the steps for the development of the four components of pediatric qFIBS (qFibrosis, qInflammation, qBallooning, and qSteatosis).

The 102 pediatric samples were divided into two groups: training group (68 samples) and validation group (34 samples), using the stratified randomization method. Using the NASH CRN scoring system as the reference standard, multiple significant parameters were selected from the training group and combined into a single index for each histological component, i.e., qFibrosis index, qInflammation index, qBallooning index, and qSteatosis index. The performance of the qFIBS indices were validated in the validation group. The details of the methods for feature selection and model construction can be found in the previous research (Figure 1) (18).

### Statistical Analysis

The Spearman non-parametric method evaluated the association between the qFIBS indices and the NASH CRN-defined pathological categories. The areas under the receiver operating characteristic curves (AUROCs) were analyzed to evaluate the diagnostic performance of the qFIBS indices for predication of the different scores of histological conponents. The optimal threshold of each modality was calculated using Youden's index. The following performance parameters were identified: sensitivity, specificity, positive predictive value (PPV), negative predictive value (PPV), positive likelihood ratio (+LR), negative likelihood ratio (–LR), and proportion of cases correctly classified. The statistical significance level was set at *P* < 0.05. All analyses were performed by MATLAB R2015a software (MathWorks, USA).

## Results

### Clinical Characteristics of the Study Participants

Table 1 shows the demographic, biochemical and histological characteristics of the 102 participants. In the training and validation groups, there were no substantial differences in the characteristics between the two groups in terms of demographic or biochemical characteristics. 10 and 15% were females (*P* = 0.58); the age range was 5–17 years (*P* = 0.58); and the mean BMI ± SD was 25.08 ± 4.29 and 24.36 ± 4.7 kg/m2 (*P* = 0.58), respectively.

**Table 1:** Laboratory and histological data of all samples.

**Variable**	**Training** **(*n* = 68)**	**Validation** **(*n* = 34)**	***P*** **values**
	***n*** **(%) or median (range)**		
**Demographics**			
Age (years)	11 (5–17)	11 (5–17)	0.52
Female (%)	7 (10.3)	5 (14.7)	0.58
BMI (kg/m2)	24.5 (19.5–41.1)	24.0 (14.5–36.4)	0.58
**Biochemical profile**			
ALT (U/L)	170 (14–805)	160 (14–397)	0.41
AST (U/L)	80 (14–543)	84 (12–459)	0.90
GGT (U/L)	58 (18–385)	49 (13–309)	0.45
ALP (U/L)	292 (77–522)	287 (100–538)	0.35
Total Bilirubin (umol/L)	9.6 (3.2–33.6)	8.7 (4.1–26.9)	0.40
Albumin (g/L)	45 (36–53)	44 (39–51)	0.18
Glucose (mmol/L)	4.9 (3.7–9.2)	4.7 (3.8–8.6)	0.11
Triglyceride (mmol/L)	1.5 (0.2–4.1)	1.6 (0.5–5.2)	0.70
Total Cholesterol (mmol/L)	4.5 (1.6–6.9)	4.2 (2.3–7.8)	0.40
HDL (mmol/L)	1.1 (0.8–1.7)	1.1 (0.6–1.5)	0.98
LDL (mmol/L)	3.0 (0.5–4.9)	2.9 (1.1–5.5)	0.18
Platelet count ( ×10^9^/L)	283 (176–483)	268 (149–386)	0.14
Histological data^*^			
**Fibrosis stage**			0.30
0			
1	6 (9)	4 (12)	
2	23 (34)	13 (38)	
3	22 (32)	12 (35)	
4	17 (25)	5 (15)	
**Lobular inflammation** **(foci/20x field)**			0.58
0			
1– <2	38 (56)	17 (50)	
2– <2–4	30 (44)	17 (50)	
3– > 4			
**Hepatocyte ballooning**			0.26
0–none			
1–mild, few	32 (47)	12 (35)	
2–moderate-marked, many	36 (53)	22 (65)	
**Steatosis grade**			0.28
0– <5%			
1– 5–33%	13 (19)	10 (29)	
2– 34–66%	21 (31)	10 (29)	
3– > 66%	34 (50)	14 (41)	

*AST, aspartate aminotransferase; ALT, alanine aminotransferase; BMI, body mass index; GGT, gamma-glutamyl transpeptidase; HDL, high-density lipoprotein; INR, international normalized ratio; LDL, low-density lipoprotein. All labs were measured while patients were fasting*.

* *NASH CRN scoring system*.

The patients were mainly distributed between stages 0 and 3 for fibrosis; grades 1 and 2 for lobular inflammation and hepatocytic ballooning; and between grades 1 and 3 for steatosis. The fibrosis stage and the grades of inflammation, ballooning and steatosis were evenly distributed between the training and validation groups.

### Training of the Pediatric qFIBS Models

The parameters for liver fibrosis, inflammation, hepatocyte ballooning, and steatosis were quantified from the SHG/TPEF images. The details of the parameters are included in the Supplementary results. During the algorithm training of discovery queue, a number of parameters were required to optimize and evaluate each histological feature in order to build the qFIBS indices. Ultimately, qFibrosis included 9 parameters, qInflammation 8 parameters, qBallooning 6 parameters, and qSteatosis just 3 parameters, in the respective models. Fibrosis and steatosis components were strongly correlated with NASH CRN (*P* < 0.001) (Supplementary Tables 1–4).

### Validation of the Pediatric qFIBS Models

**Figure 2:**
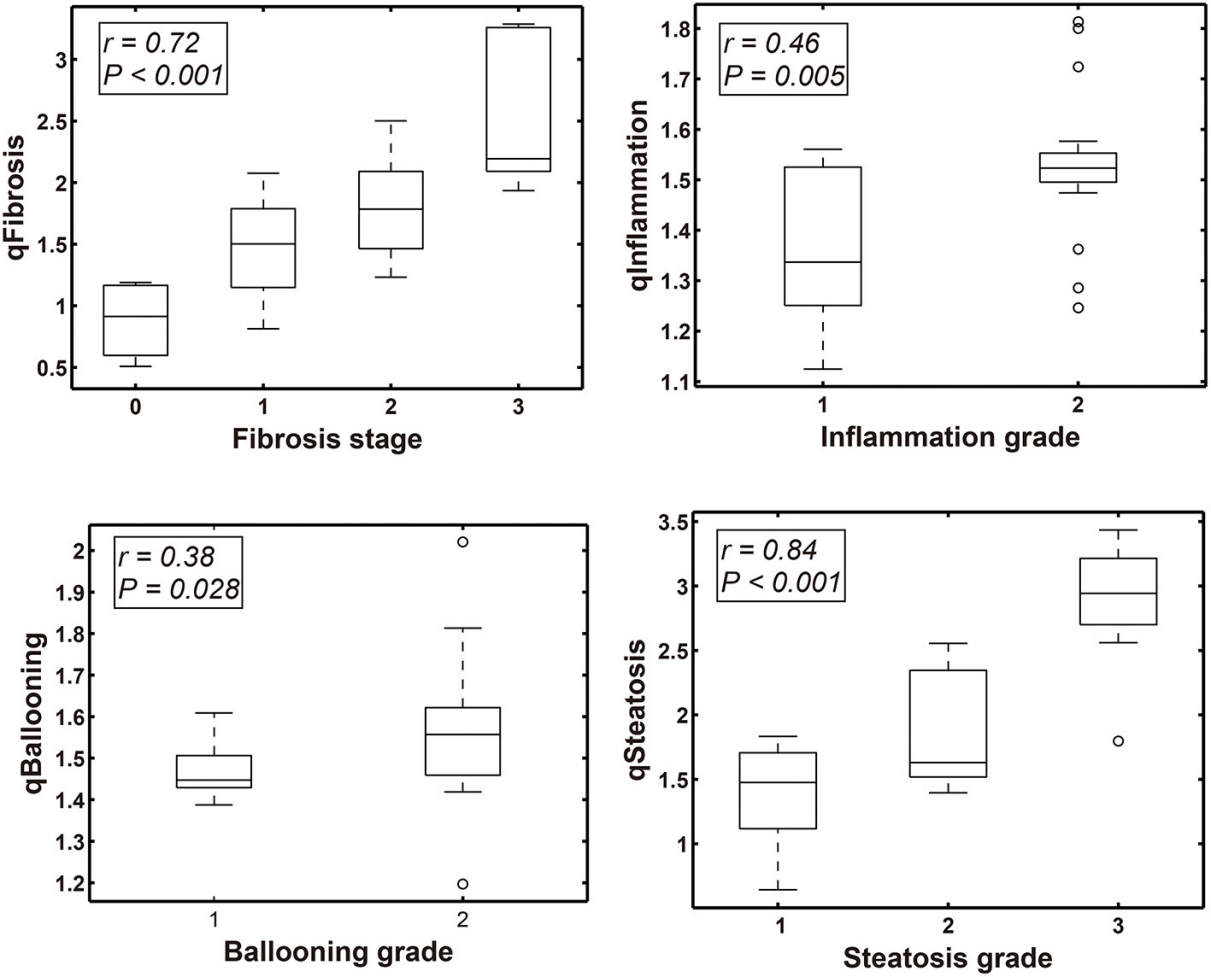
Box-Whisker plots show the correlation between qFIBS indices (qFibrosis, qInflammation, qBallooning, and qSteatosis) and Non-alcoholic Steatohepatitis Clinical Research Network (NASH CRN) score component in the pediatric validation group.

In the validation cohort (*n* = 34 cases), qFibrosis correlated strongly with histological fibrosis stage (*r* = 0.72) and could accurately differentiate fibrosis stages with an area under the curve (AUC) > 0.85 (0.85–0.95) [95% confidence interval (CI), 0.722–1.000]. Similarly, qSteatosis exhibited the highest level of correlation with the histological steatosis score (*r* = 0.84) and performed especially well, differentiating grades of steatosis with very high levels of accuracy, with an AUC > 0.90 (0.90–0.98) (95% CI, 0.777–1.000; sensitivity, 0.71 and 0.93; specificity, 0.90 and 0.90; +LR, 7.08 and 9.29; –LR, 0.32 and 0.08). qInflammation increased with grade (*r* = 0.46) and could differentiate grades 1 and 2 of inflammation (AUC = 0.77; 95% CI, 0.609–0.927; sensitivity, 0.83 and specificit,y 0.53). Although qBallooning showed a weak correlation with ballooning, it could distinguish between grades 1 and 2 of ballooning (*r* = 0.38) (AUC = 0.73; 95% CI, 0.554–0.895; sensitivity 0.36 and specificity 0.92) (Figure 2, Table 2).

**Table 2:** Performance of qFIBS models for pediatric validation group.

	**AUROC**	**95%CI**	***p*** **value**	**Cut-off**	**Sensitivity**	**Specificity**	**PPV**	**NPV**	**+LR**	**–LR**	**%Cases correctly classified**
**qFibrosis**											
0 vs. 1/2/3	0.95	0.873–1.000	0.004	1.0795	93%	50%	93%	40%	1.87	0.13	85%
0/1 vs. 2/3	0.85	0.722–0.974	0.001	1.4501	88%	59%	67%	77%	2.14	0.20	71%
0/1/2 vs. 3	0.92	0.817–1.000	0.003	2.1745	60%	90%	50%	93%	5.80	0.45	85%
**qInflammation**											
1 vs. 2	0.77	0.609–0.927	0.007	1.4016	83%	53%	65%	75%	1.77	0.31	68%
**qBallooning**											
1 vs. 2	0.73	0.554–0.895	0.031	1.6044	36%	92%	89%	44%	4.36	0.69	56%
**qSteatosis**											
1 vs. 2/3	0.90	0.777–0.999	<0.001	1.8836	71%	90%	100%	59%	7.08	0.32	79%
1/2 vs. 3	0.98	0.933–1.000	<0.001	2.445	93%	90%	93%	95%	9.29	0.08	94%

*AUROC, area under the receiver operating characteristic; CI, confidence interval; PPV, Positive predictive value; NPV, negative predictive value; +LR, Positive Likelihood ratio; -LR, Negative Likelihood ratio*.

### Validation of the Adult qFIBS Models in a Pediatric Cohort

To demonstrate whether the qFIBS model for adult NASH can be directly applied to the pediatric NASH group, we analyzed the adult qFIBS models in pediatric NASH by using all 102 pediatric NASH biopsies as the validation group. qSteatosis showed a high correlation with steatosis (*r* = 0.81, *P* < 0.001); it was able to distinguish different grades of steatosis (AUROCs, 0.91 and 0.95; sensitivity, 0.91 and 0.90; specificity, 0.61 and 0.81; +LR, 2.33 and 4.84; –LR, 0.15 and 0.13). The qFIBS correlation with fibrosis (*r* = 0.60, *P* < 0.001) was weaker; it demonstrated high AUROC values [qFibrosis (AUC) >0.80 (0.80–0.82)] and could distinguish different stages of fibrosis. qFIBS showed no correlation with ballooning (*r* = 0.17, *P* = 0.094) and inflammation (*r* = 0.08, *P* = 0.41) but could distinguish different grades of ballooning (AUROC 0.61, sensitivity 0.48, and specificity 0.61) and inflammation (AUROC 0.55, sensitivity 062, and specificity 0.49). The results show that qFibrosis and qSteatosis are the most sensitive and applicable, while qBallooning and qInflammation are not as applicable to the pediatric qFIBS model (Supplementary Figure 1, Supplementary Table 5).

## Discussion

NAFLD/NASH is becoming increasingly recognized as an important health problem in the pediatric population. Because there is no established effective therapy, there is an urgent clinical need for accurate diagnosis and patient management. Routine non-invasive methods, such as blood tests and imaging, are useful to identify pediatric NAFLD. ALT and γ-glutamyl transpeptidase (GGT) levels correlate well with liver biopsy changes in children with NASH (25). However, studies on the use of these laboratory panels to determine NASH progression are still ongoing. Although MRE and magnetic resonance imaging-derived proton density fat fraction (MRI-PDFF) show some of the best accuracies in assessing fibrosis and steatosis in the adult NAFLD population, the currently available literature in pediatric NAFLD populations suggests good results but a lower sensitivity (26). Most studies of biomarkers for NASH in children have examined a limited number of biomarkers in small cohorts without evaluation of NASH histology (27, 28). Therefore, the current data from imaging modalities, blood tests or biomarkers are not sufficiently sensitive for NAFLD diagnosis.

Liver biopsy remains the reference standard for diagnosing and staging NAFLD/NASH. Several studies have reported the use of advanced technology to improve diagnostic accuracy, such as digitized images of tissue sections (17–21). However, there are no reports of digital imaging of liver biopsy for pediatric NAFLD. Our previous research (18) established a novel SHG/TPEF-based quantitative evaluation tool termed “qFIBS” for the evaluation of the four cardinal histological features of NASH in adult patients. In this study, we first tested whether the adult qFIBS model could be applied to pediatric NASH patients.

We optimized and evaluated each histological feature to develop four independent component models of pediatric qFIBS, and demonstrated a high degree of concordance for qFibrosis and qSteatosis with the relevant NASH CRN features, whereas the concordances for ballooning and inflammation were lower. Notably, with the lower concordances of ballooning and lobular inflammation, there was higher interobserver variability in pathological assessment (13, 14); this inconsistency of the reference standard might have an inherent effect on the development of alternative approaches to assess liver histology features in NAFLD.

qSteatosis had the best performance (AUC ≥ 0.90), likely because it is more easily assessed than the other three features. Similar to our results, Munsterman et al. (29) developed a digital image analysis (DIA) algorithm to find potential steatotic hepatocytes with an AUC of 0.970 (95% CI 0.968–0.973). A relatively good performance was observed with fibrosis stage (AUC ≥ 0.85) since collagen signals are more easily captured by SHG based on the unique architectural features of collagen. SHG can automate the quantification of fibrosis, as shown in a series of studies to assess liver fibrosis; qFibrosis has been reported to be very accurate in evaluating the different stages of fibrosis in chronic hepatitis B and NAFLD patients (30, 31). Inflammation and ballooning features achieved less impressive results (AUC: 0.77 and 0.73, respectively), which may suggest a greater degree of difficulty in training the features compared with steatosis and fibrosis.

Several computer-assisted digital image studies have attempted to improve the diagnostic accuracy and have reported a correlation between the clinical staging score and pathological features obtained by image analysis. Gawrieh et al. (17) developed an automated software for detecting and quantifying hepatic fibrosis from liver biopsy samples obtained from NAFLD patients and reported a significant correlation between the artificial intelligence-based system and the human pathologist's fibrosis stage score. Forlano et al. (21) created complete automated software for diagnosing and quantifying the main histological features from NAFLD liver biopsy samples and for identifying histological characteristics of NAFLD with inter- and intraobserver agreement levels that ranged between 0.95 and 0.99. However, these investigators have relied largely on digitized images of stained biopsy slides. The stained liver samples might involve image sharpening with noise removal, segmentation, and artifact removal depending on the image quality. In our study, the SHG/TPEF system focused on unstained slides and avoided the shortcomings of stained sections.

When we analyzed the adult qFIBS model in the pediatric NASH cohort by using all pediatric NASH biopsies as the validation group, the results showed that qFibrosis and qSteatosis were the most suitable; qBallooning and qInflammation were not applicable to the pediatric qFIBS model (ballooning: *r* = 0.17; inflammation: *r* = 0.08). This discrepancy may be related to the similarities and differences between pediatric and adult NAFLD (24). Children can not only have “adult-type” zone 3 NASH, with or without ballooned hepatocytes, but also a “pediatric” zone 1 NASH characterized by periportal or panacinar steatosis, portal fibrosis, and primarily portal inflammation. Therefore, in this study, when we tested and validated only the cohort of children with pediatric pattern NASH, the overall performance of the qFIBS was better.

Our research suggested that the pediatric qFIBS could be used for quantification of the main NASH histological features, but the analysis has some limitations. First, the proposed qFIBS was established using pediatric data and needs to be further validated in larger cohorts, especially in patients from different regions, as has been done with the adult qFIBS. The available automated qFIBS methods mainly depend on international collaboration to set up a standardization and validation platform for full applicability of qFIBS to pediatric NASH. Second, our study only included steatohepatitis diagnosis in pediatric NASH patients, so no cases of grade 0 inflammation, ballooning or steatosis were analyzed. There were also no instances of stage 4 fibrosis. In children, NAFLD has been reported to run a milder course with a very low incidence of cirrhosis (32, 33). Hence, the present data showed that qFIBS could not assess inflammation and ballooning properly. The pediatric NAFLD patient population needs to be expanded with more instances of inflammation, ballooning, steatiosis, and cirrhosis to perform as diagnostic as well as severity assessment tool for NASH. Third, the epidemiological studies have shown that zone 1 NASH is more common in younger children, obese boys, and children of non-White origin, while adolescents tend to develop zone 3 NASH or adult-type features (34, 35). It would be interesting to analyze the liver histology of children with NAFLD according to different age ranges.

## Conclusion

The results of this study demonstrate that it is feasible to establish a supervised machine learning system—qFIBS—to automatically quantify the four main histological features needed for the pediatric NASH phenotype. qFIBS is very precise and highly reproducible and is particularly capable of differentiating the histological grades of steatosis and fibrosis. Compared with the semiquantitative scores used to measure NAFLD activity, these findings produce more accurate and desirable continuous measurements to analyze NAFLD patient's therapy response in clinical trials or patient care. Our group is currently working to extend these methods to artificial intelligence (AI) / machine learning methods to further improve the accuracy of liver biopsy interpretation. The current research lays the foundation for our group and other groups to provide more accurate drug-based histopathological analysis of liver biopsy, and finally strengthened patient care.

## Data Availability Statement

The original contributions presented in the study are included in the article/Supplementary Material, further inquiries can be directed to the corresponding author/s.

## Ethics Statement

The studies involving human participants were reviewed and approved by the Ethics Committees of Peking University People's Hospital and the Fifth Medical Center of PLA General Hospital (No. 2017PHB133-01). Written informed consent to participate in this study was provided by the participants' legal guardian/next of kin. Written informed consent was obtained from the individual(s), and minor(s)' legal guardian/next of kin, for the publication of any potentially identifiable images or data included in this article.

## Author Contributions

AW, J-MZ, FL, and LW conceived the study design. FL and LW developed the method plan. FL, LW, S-HL, X-XW, X-HL, H-YR, RH, NW, and J-MZ performed data collection and interpretaion. AW, J-MZ, and WL evaluated liver biopsy pathology. FL performed all the data management and statistical analysis and drafted the first manuscript. H-YR, LW, J-MZ, and AW gave the critical revision. All authors contributed to the article and approved the submitted version.

## Funding

This work was supported by grants from the China National Science and Technology Major Project for Infectious Diseases Control during the 13th Five-Year Plan Period (2018ZX09201002-001-005), National Key R&D Program of China (2016YFE0116800), and National Natural Science Foundation of China (NSFC) (81870407 and 82170584).

## Conflict of Interest

LW is a consultant for AbbVie, Allergan/Tobira, Novartis Pharma AG, Pfizer Ltd., Galmed, Gilead, HistoIndex, and Janssen. His institution has received research grant funding from AbbVie, BMS, and Gilead. Y-YR was employed by HistoIndex Pte Ltd. The remaining authors declare that the research was conducted in the absence of any commercial or financial relationships that could be construed as a potential conflict of interest.

## Publisher's Note

All claims expressed in this article are solely those of the authors and do not necessarily represent those of their affiliated organizations, or those of the publisher, the editors and the reviewers. Any product that may be evaluated in this article, or claim that may be made by its manufacturer, is not guaranteed or endorsed by the publisher.
