# Supplementary material for: Developing a New qFIBS Model Assessing Histological Features in Pediatric Patients With Non-alcoholic Steatohepatitis
Source: Front Med (Lausanne). 2022 Jun 27;9:925357. doi: 10.3389/fmed.2022.925357 (PMC9271828; doi:10.3389/fmed.2022.925357)
Supplement: Supplementary file 1 [file Data_Sheet_1.docx]

Supplementary Material

# Supplementary Table 1: Selected parameters (n=9) for qFibrosis model.

| NO. | Parameter | Description | r | P |
| --- | --- | --- | --- | --- |
| 1 | FiberMWidthAll | The total maximal width of fibers at overall region | 0.42 | <0.001 |
| 2 | FiberLengthAggAll | The total length of aggregated fibers at overall region | 0.45 | <0.001 |
| 3 | FiberPerimeterDisAll | The total perimeter of distributed fibers at overall region | -0.11 | 0.372 |
| 4 | #ThinFiberCV | The number of thin fibers at central vein region | -0.06 | 0.652 |
| 5 | #ShortFiberDisCV | The number of short and distributed fibers at central vein region | -0.06 | 0.644 |
| 6 | AggAreaPT | The area of aggregated fibers at portal tract region | 0.71 | <0.001 |
| 7 | FiberAWidthAggPT | The total average width of aggregated fibers at portal tract region | 0.67 | <0.001 |
| 8 | #ThickFiberAggPT | The number of thick and aggregated fibers at portal tract region | 0.69 | <0.001 |
| 9 | FiberAWidthDisPT | The total average width of distributed fibers at portal tract region | 0.14 | 0.260 |

# Supplementary Table 2: Selected parameters (n=8) for qInflammation model.

| NO. | Parameter | Description | r | P |
| --- | --- | --- | --- | --- |
| 1 | %AreaLT | The percentage of inflammation (TPEF) at lobular region | -0.06 | 0.601 |
| 2 | #AInflammationPTS | The average number of inflammation (SHG) per unit area at portal tract region | 0.03 | 0.778 |
| 3 | #InflammationLCVA | The number of inflammation (ALL) at lobular region | 0.20 | 0.096 |
| 4 | #InflammationA/%AreaA | The ratio of #InflammationA to %AreaA | 0.03 | 0.793 |
| 5 | %AreaPTT/#InflammationPTT | The ratio of %AreaPTT to #InflammationPTT | -0.18 | 0.152 |
| 6 | #InflammationPTS/  #AInflammationPTS | The ratio of #InflammationPTS to #AInflammationPTS | 0.20 | 0.094 |
| 7 | #InflammationLCVA/  #AInflammationLCVA | The ratio of #InflammationLCVA to #AInflammationLCVA | 0.27 | 0.029 |
| 8 | #AInflammationLCVA/  #InflammationLCVA | The ratio of #AInflammationLCVA to #InflammationLCVA | 0.07 | 0.586 |

# Supplementary Table 3: Selected parameters (n=6) for qBallooning model.

| NO. | Parameter | Description | R | P |
| --- | --- | --- | --- | --- |
| 1 | MaxArea/#BCell | The ratio of MaxArea to #BCell at overall region | -0.09 | 0.465 |
| 2 | MaxDensityBCell/#BCell | The ratio of MaxDensityBCell to #BCell at overall region | -0.19 | 0.130 |
| 3 | MaxAreaLobular/#BCellLobular | The ratio of MaxArea to #BCell at lobular region | -0.07 | 0.576 |
| 4 | MaxAreaPT/MaxArea | The ratio of MaxAreaPT to MaxArea | 0.14 | 0.251 |
| 5 | AreaBCellLobular/AreaBCell | The ratio of AreaBCellLobular to AreaBCell | -0.31 | 0.010 |
| 6 | AverageDensityBCellLobular/  AverageDensityBCell | The ratio of AverageDensityBCellLobular to AverageDensityBCellPT | -0.30 | 0.014 |

# Supplementary Table 4: Selected parameters (n=3) for qSteatosis model.

| NO. | Parameter | Description | r | P |
| --- | --- | --- | --- | --- |
| 1 | %MacroArea | The percentage of macrovesicular steatosis at overall region | 0.82 | <0.001 |
| 2 | #BubblePT | The number of fat vacuoles at portal tract region | 0.06 | 0.619 |
| 3 | #MicroBubbleCV | The number of micro fat vacuoles at central vein region | 0.06 | 0.645 |


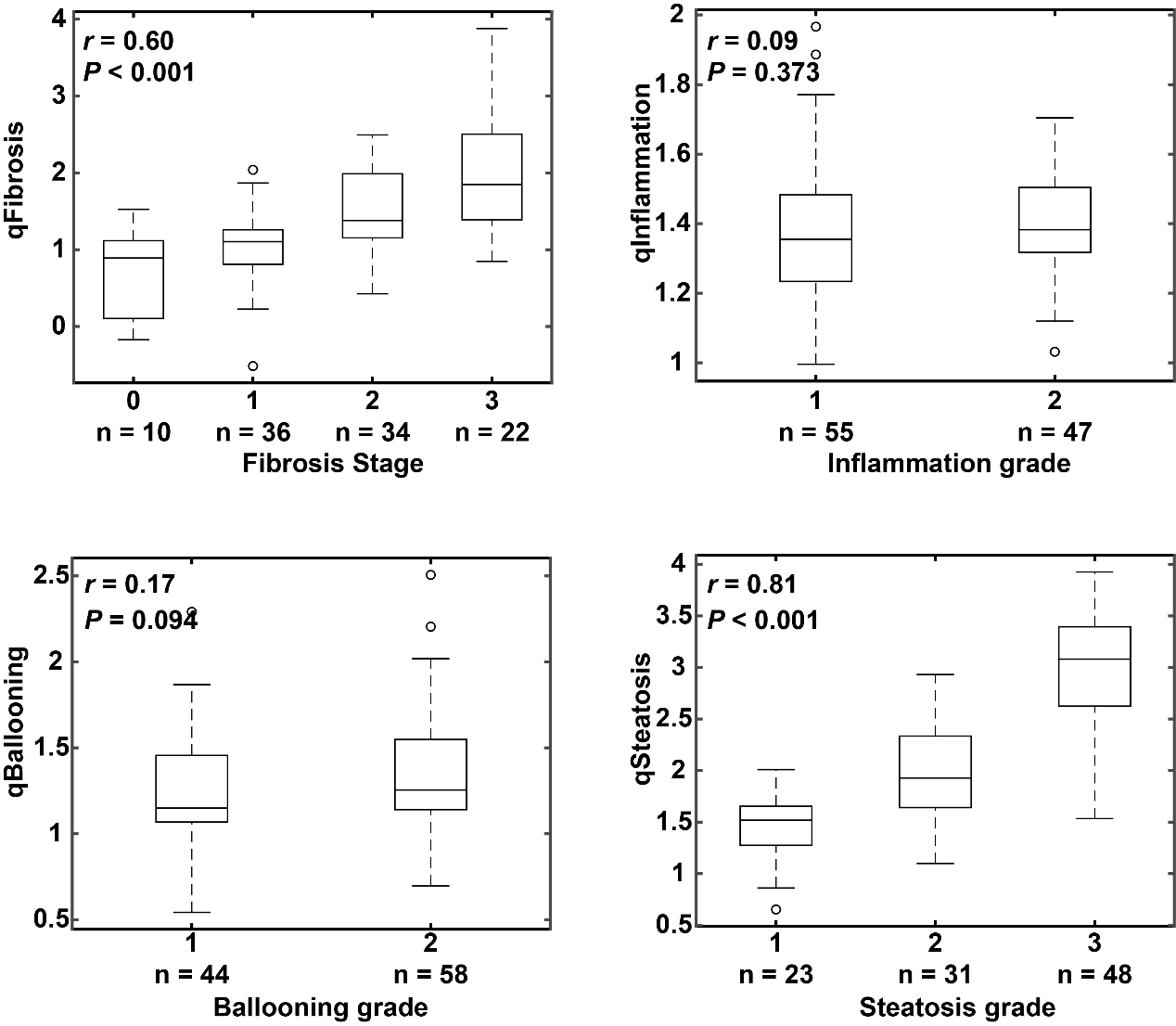


**Supplementary Figure 1**. Box-Whisker plots of values of each qFIBS component distribution relative to the NASH CRN Score component category in the pediatric Validation Group according to adult qFIBS model.

# Supplementary Table 5: Performance of qFIBS adult models for pediatric liver samples of NASH

|  | **AUROC** | **95%CI** | ***P* value** | **Cut-off**  **(qFIBS)** | **Sensitivity*** | **Specificity*** | **PPV** | **NPV** | **+LR** | **-LR** | **%Cases correctly classified** |
| --- | --- | --- | --- | --- | --- | --- | --- | --- | --- | --- | --- |
| ***qFibrosis*** |  |  |  |  |  |  |  |  |  |  |  |
| **F0 vs F1/2/3** | 0.80 | 0.650-0.939 | 0.002 | 0.761 | 91% | 40% | 93% | 33% | 1.52 | 0.22 | 86% |
| **F0/1 vs F2/3** | 0.82 | 0.745-0.902 | <0.001 | 0.882 | 95% | 33% | 63% | 83% | 1.40 | 0.16 | 67% |
| **F0/1/2 vs F3** | 0.80 | 0.693-0.906 | <0.001 | 1.491 | 64% | 75% | 42% | 88% | 2.55 | 0.48 | 74% |
| ***qInflammation*** |  |  |  |  |  |  |  |  |  |  |  |
| **1 vs 2** | 0.55 | 0.439-0.664 | 0.370 | 1.357 | 62% | 49% | 52% | 61% | 1.21 | 0.78 | 56% |
| ***qBallooning*** |  |  |  |  |  |  |  |  |  |  |  |
| **1 vs 2** | 0.60 | 0.485-0.710 | 0.094 | 1.266 | 48% | 61% | 64% | 48% | 1.25 | 0.84 | 55% |
| ***qSteatosis*** |  |  |  |  |  |  |  |  |  |  |  |
| **1 vs 2/3** | 0.91 | 0.861-0.968 | <0.001 | 1.572 | 91% | 61% | 90% | 68% | 2.33 | 0.15 | 85% |
| **1/2 vs 3** | 0.95 | 0.903-0.990 | <0.001 | 2.21 | 90% | 81% | 81% | 90% | 4.84 | 0.13 | 85% |

Abbreviations: AUROC, area under the receiver operating characteristic; CI, confidence interval; PPV, Positive predictive value; NPV, negative predictive value; +LR, Positive Likelihood ratio; -LR, Negative Likelihood ratio

*Sensitivity and specificity were calculated based on the cut-off values published in qFIBS paper (ref..18)
